# Association Between Low-Density Lipoprotein Cholesterol and Platelet Distribution Width in Acute Ischemic Stroke

**DOI:** 10.3389/fneur.2021.631227

**Published:** 2021-03-05

**Authors:** Jian Yuan, Jian Cai, Pei Zhao, Nan Zhao, Rong-Hua Hong, Jie Ding, Jin Yang, Qing-Lei Fan, Jian Zhu, Xia-Jun Zhou, Ze-Zhi Li, De-Sheng Zhu, Yang-Tai Guan

**Affiliations:** ^1^Department of Neurology, Baoshan Branch, Renji Hospital, School of Medicine, Shanghai Jiaotong University, Shanghai, China; ^2^Department of Neurology, Renji Hospital, School of Medicine, Shanghai Jiaotong University, Shanghai, China

**Keywords:** acute ischemic stroke, platelet distribution width, low-density lipoprotein, oxidative stress, multivariate analysis

## Abstract

**Objective:** Elevated low-density lipoprotein cholesterol (LDL-C) is an established risk factor for ischemic stroke; however, whether LDL-C affects the platelet deformation function in the peripheral blood circulation in patients with acute ischemic stroke (AIS) is unknown. The present study aimed to investigate the relationship between LDL-C and platelet distribution width (PDW) in AIS patients.

**Methods:** We conducted a cross-sectional hospitalized-based study of consecutive 438 patients with AIS within 24 h. Blood samples were collected upon admission and prior to drug administration, and LDL-C and PDW (a parameter that reflects the heterogeneity of platelet volume) were assessed. The relationship between LDL-C and PDW were analyzed by linear curve fitting analyses. Crude and adjusted beta coefficients of LDL-C for PDW with 95% confidence intervals were analyzed using multivariate-adjusted linear regression models.

**Results:** The PDW was significantly higher in the high LDL-C group compared with those in the normal LDL-C group (16.28 ± 0.37 fl vs. 16.08 ± 0.37 fl, *p* < 0.001). Adjusted smoothed plots suggested that there are linear relationships between LDL-C and PDW, and the Pearson's correlation coefficient (95%) was 0.387 (0.304–0.464, *p* < 0.001). The beta coefficients (95% CI) between LDL-C and PDW were 0.15 (0.12–0.18, *p* < 0.001) and 0.14 (0.11–0.18, *p* < 0.001), respectively, in AIS patients before and after adjusting for potential confounders.

**Conclusion:** Our study suggested that the elevated LDL-C level was related to increased PDW among AIS patients.

## Introduction

Acute ischemic stroke (AIS), a neurological deficit syndrome caused by necrosis and softening of brain tissue, is a common cause of morbidity and mortality worldwide, and its incidence was 60–70% among all strokes (1). Elevated low-density lipoprotein cholesterol (LDL-C) has been considered an independent risk factor for AIS. LDL-C is the main cholesterol-carrying lipoprotein in the body, accounting for ~70% of circulating cholesterol (2). Its physiological function is to transport the endogenous cholesterol synthesized in the liver to all tissues of the body, while elevated LDL-C will increase the amount of cholesterol transported to all parts of the body, leading to atherosclerosis. Studies have revealed that elevated LDL-C is associated with the development of atherosclerotic cerebrovascular diseases and related mortality (3–5). Previous studies on the mechanism of AIS induced by LDL-C mostly focus on vascular inflammation and atherosclerosis. Few studies have reported on the relationship between LDL-C and hemodynamics and composition in the peripheral blood circulation.

The occurrence of cerebral infarction is closely related to the vascular wall, blood composition and hemodynamics, and the blood composition factors include the number and morphological changes of platelets, red blood cells, and white blood cells (6). Platelet distribution width (PDW) is a parameter reflecting the volume heterogeneity of peripheral blood platelet, which is expressed by the variation coefficient of measured platelet volume. The increase of PDW indicates the great difference in platelet size, which is common in hematological diseases and thrombotic diseases (7). Previous studies have observed the morphologic changes and reactivity enhancement of platelets in myocardial infarction and other peripheral vascular thrombotic diseases (8, 9). A recent study showed that PDW was an important risk factor for stroke, and it could be a new biomarker for predicting stroke (10).

Based on current evidence, whether LDL-C affects the platelet deformation function in peripheral blood circulation and thus causes ischemic stroke needs to be further investigated. Thus, we conducted this cross-sectional study, aiming to assess the association between LDL-C and PDW in the AIS population. To our knowledge, no study has previously reported on the association between LDL-C levels and PDW in the AIS population.

## Materials and Methods

### Ethics

This study was performed according to the principles of the Declaration of Helsinki, and was approved by the ethics committee of Baoshan Branch, Renji Hospital, School of Medicine, Shanghai Jiaotong University, Shanghai, China. We obtained informed consent from all patients or their immediate family members prior to sample collection.

### Design

This was a cross-sectional study, designed to explore the correlation between LDL-C and PDW in the AIS population. Consecutive patients with AIS were enrolled in our study from Baoshan Branch of Renji Hospital in China from January 1, 2018, and September 31, 2019, and patient data were recorded in the Stroke Registry Database of the hospital.

### Study Subjects

Patients were diagnosed with AIS according to the criteria defined by the World Health Organization (11). The inclusion criteria were (1) acute onset of ischemic stroke within 24 h; (2) ischemic stroke symptoms and signs that can be clinically evaluated; (3) confirmation by computed tomography (CT) or magnetic resonance imaging (MRI) of the brain within 24 h after admission, follow-up CT, or MRI was performed within 14 days of admission or in any case of neurological deterioration; and (4) aged ≥40 years.

Patients were excluded according to the following exclusion criteria: (1) intracerebral hemorrhage; (2) transient ischemic attack; (3) malignancies; (4) leukemia, megaloblastic anemia, post-splenectomy, giant platelet syndrome, primary thrombocytopenia, and aplastic anemia; (5) acute myocardial infarction and cardiac valvulopathy; and (6) data were not available for review, including unintegrated clinical and laboratory data.

### Clinical and Laboratory Data

The data for demographic features, medical history [hypertension, diabetes, coronary heart disease (CHD), and atrial fibrillation], and medication used before admission (antihypertensive drugs, lipid lowering drugs, antidiabetic drugs, anticoagulant drugs, and antiplatelet drugs) were collected upon admission via in-person interviews with the patients or their family members.

Fasting venous blood samples were collected upon admission and prior to drug administration, such as intravenous tissue plasminogen activator, or any intraarterial revascularization procedure in the emergency room. Blood specimens were collected using coagulation-promoting vacuum tubes to detect the level of LDL-C, which were measured with commercially available quantitative test kit obtained from Biotechnology Co., Ltd (Shanghai, China). Intra- and interassay coefficients of variation were 5 and 10%, respectively. The detection values ranged from 0.05 to 11.60 mmol/L for LDL-C. The LDL-C reference value in our laboratory was <3.1 mmol/L. Another blood specimen was collected using EDTA vacuum tube to detect the level of PDW, which was measured by XFA6100 automatic hematology analyzer. Intra- and interassay coefficients of variation were 4 and 10%, respectively. The normal range of PDW ranged between 9.8 and 16.1 fl.

Blood samples were also collected to measure routine blood indicators [red blood cell (RBC) count, red blood cell distribution width, platelet count, white blood cell (WBC) count, neutrophil count, and lymphocyte count], blood biochemical indicators (levels of alanine aminotransferase, total bilirubin, uric acid, urea, creatinine, fasting blood sugar, glycosylated hemoglobin, homocysteine, and erythrocyte sedimentation rate), blood lipid index [levels of high density lipoprotein cholesterol (HDL-C), LDL-C, triglyceride, apolipoprotein A1, and apolipoprotein B], and clotting index (D-Dimer, prothrombin time, partial thromboplastin time, and thrombin time). All the above determinations were performed in the hospital's laboratory by individuals blinded to the clinical data.

### Groups

The patients in our study were grouped according to two criteria. (1) First, in baseline characteristics analysis, groups were organized by LDL-C level. The normal range of LDL-C was <3.1 mmol/L; thus, high LDL-C was identified when its value was ≥3.1 mmol/L, and included patients were categorized into a group with normal LDL-C level (0–3.1 mmol/L) and a group with high LDL-C level (≥3.1 mmol/L). When levels of PDW were compared, patients were also categorized into the T1, T2, and T3 groups according to the LDL-C tertile levels. (2) Second, groups were organized based on the clinical normal reference value of indicators in the hierarchical analysis. The normal range of total bilirubin was <22.25 μmol/L, and those of uric acid, fasting glucose, and triglyceride were <426 μmol/L, 6.1 mmol/L, and 1.7 mmol/L, respectively.

### Statistical Analysis

Baseline characteristics of participants are presented by LDL-C level. Categorical variables were presented as counts and percentages and were analyzed by Fisher's exact tests or Chi-square tests. Continuous variables were reported as the means and standard deviations for data of normal distribution, which were analyzed by *t*-tests, and they were reported as medians and interquartile ranges for data of abnormal distribution, which were analyzed by Mann–Whitney *U*-tests. The association between LDL-C and PDW was assessed by linear curve fitting analyses and multiple linear regression analysis. Both non-adjusted and multivariate adjusted models were applied, and interaction and stratified analyses were conducted. Statistical analyses were performed using Statistical Package of the Social Sciences Software version 24.0 (SPSS, Chicago, IL, USA), and statistical graphics were generated using GraphPad PRISM 6 (Graph Pad Software Inc., San Diego, CA, USA). The level of significance was set with a two-tailed *p*-value of <0.05.

## Results

### Baseline Characteristics

**Figure 1:**
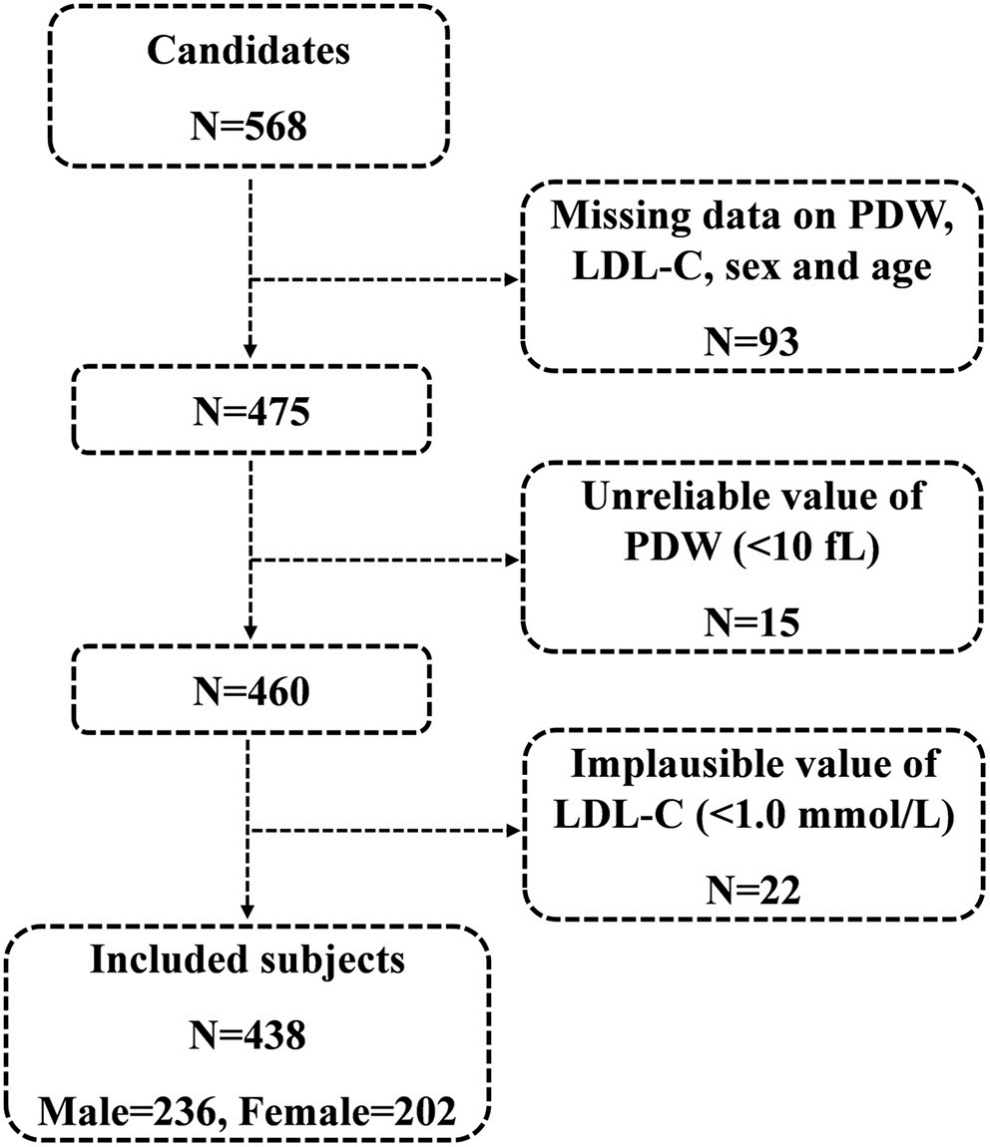
A flowchart of the study.

A total of 568 consecutive candidates were recruited for the study at the time of the final survey on July 31, 2019. Among these candidates, those who had missing data related to PDW, LDL-C, sex, and age were excluded from the eligible candidates for this study (*n* = 93). Those with unreliable values of PDW (<10 fl) (*n* = 15) and those with implausible values of LDL-C (<1.0 mmol/L) (*n* = 22) were also excluded from the pool of eligible candidates for this study. As a result, a total of 438 subjects were included in the final analyses. A flowchart of the study is shown in Figure 1.

**Figure 2:**
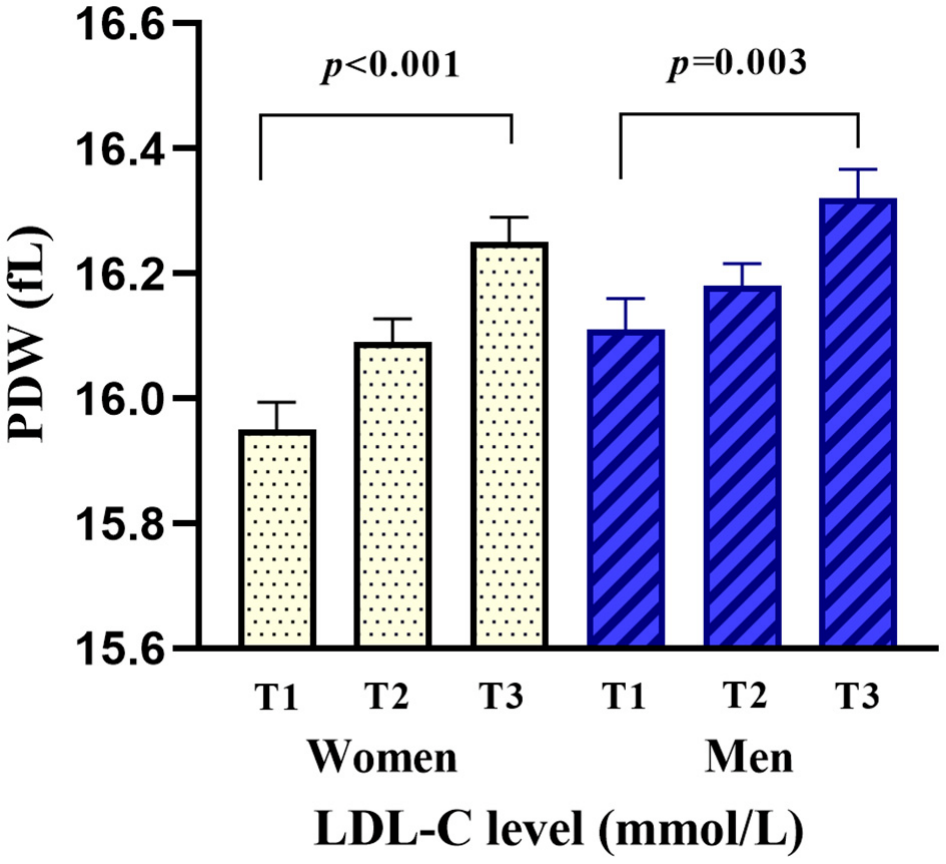
Comparison of PDW by the tertiles of LDL-C levels and sex. PDW was significantly elevated with the increased tertiles of LDL-C levels in women (*p* < 0.001) and men (*p* = 0.003).

Among 438 study subjects, women accounted for 46.12% (*n* = 202) and men accounted for 53.88% (*n* = 236). The age of the enrolled subjects ranged from 40 to 99 years (women, 49–99 years; men, 40–91 years) with a mean age of 73.07 ± 10.83 years (women, 76.47 ± 10.24 years; men, 70.17 ± 10.49 years). The disease duration before admission ranged from 0.5 to 24 h, and the value was abnormally distributed with a median and interquartile range of 6.5 (3.5–9.5) h. The LDL-C ranged from 1.01 to 6.15 mmol/L, and the PDW ranged from 15.2 to 17.5 fl. The mean PDW was significantly higher in the high LDL-C group than that it was in the normal LDL-C group (16.15 ± 0.38 vs. 16.08 ± 0.37 fl, *p* < 0.001), and hierarchical analysis by sex showed a significant difference in women (*p* < 0.001) and men (*p* = 0.003). Higher LDL-C levels were associated with increased levels of PDW, triglyceride, apolipoprotein B, and uric acid at baseline. The baseline characteristics of the included patients are shown in Table 1. The mean serum PDW levels were 16.03 ± 0.40, 16.14 ± 0.32, and 16.29 ± 0.37 fl in the first (T1), second (T2), and third (T3) RDW-SD tertiles for all patients, respectively, and there was a significant difference among the three groups (*p* < 0.001). Hierarchical analysis by sex also showed that PDW was significantly elevated with the increased tertiles of LDL-C levels (Supplementary Table 1, Figure 2).

**Table 1:** Baseline characteristics of participants by LDL-C level.

**Index**	**Total patients (*N* = 438)**	**Normal LDL-C group (*N* = 276)**	**High LDL-C group (*N* = 162)**	***p-*value**
**Basic information**				
Sex (men) (%)	236 (53.88)	145 (52.54)	91 (56.17)	0.461
Age (years)	73.07 ± 10.83	73.21 ± 10.65	72.83 ± 11.16	0.719
Disease duration (h)	6.50 (3.50–9.50)	6.25 (3.50–9.12)	6.75 (3.50–11.00)	0.694
**OCSP classification**				
TACI (%)	49 (11.19)	30 (10.87)	19 (11.73)	0.920
PACI (%)	133 (30.36)	81 (29.35)	52 (32.10)	0.617
POCI (%)	82 (18.72)	55 (19.93)	27 (16.67)	0.471
LACI (%)	174 (39.73)	110 (39.85)	64 (39.50)	1.000
**Medical history**				
Hypertension (%)	368 (84.02)	226 (81.88)	142 (87.65)	0.112
Diabetes (%)	153 (34.93)	96 (34.78)	57 (35.19)	0.932
CHD (%)	123 (28.08)	72 (26.09)	51 (31.48)	0.225
Atrial fibrillation (%)	14 (3.20)	10 (3.62)	4 (2.47)	0.586
**Blood biochemical indicators**				
ALT (U/L)	17.05 (12.12–24.20)	16.30 (12.00–23.90)	17.85 (12.22–24.37)	0.337
Total bilirubin (μmol/L)	11.85 (8.60–15.97)	11.50 (8.57–15.60)	12.10 (8.83–16.43)	0.492
Uric acid (μmol/L)	291.31 ± 109.11	283.34 ± 106.70	304.88 ± 112.13	0.046
Urea (mmol/L)	5.50 (4.50–6.70)	5.50 (4.40–6.53)	5.55 (4.62–6.88)	0.261
Creatinine (μmol/L)	80.05 ± 39.04	77.84 ± 38.62	83.83 ± 39.58	0.121
Fasting glucose (mmol/L)	6.16 ± 2.20	6.06 ± 2.03	6.33 ± 2.47	0.219
Homocysteine (μmol/L)	16.96 ± 12.28	16.75 ± 11.81	17.34 ± 13.07	0.626
ESR (mm/h)	22.00 (11.00–30.00)	23.00 (12.00–30.25)	20.00 (10.00–28.75)	0.208
**Blood lipid index**				
HDL-C (mmol/L)	1.25 ± 0.35	1.25 ± 0.37	1.27 ± 0.30	0.489
LDL-C (mmol/L)	2.84 ± 0.98	2.24 ± 0.53	3.87 ± 0.67	<0.001
Triglyceride (mmol/L)	1.16 (0.83–1.56)	1.05 (0.80–1.45)	1.31 (1.00–1.78)	<0.001
Apolipoprotein A1 (g/L)	1.08 ± 0.24	1.06 ± 0.27	1.10 ± 0.18	0.107
Apolipoprotein B (g/L)	0.92 ± 0.33	0.81 ± 0.35	1.12 ± 0.17	<0.001
**Blood routine indicators**				
RBC (10^12^/L)	4.39 ± 0.63	4.36 ± 0.62	4.44 ± 0.64	0.234
RDW (fl)	42.02 ± 2.84	42.06 ± 2.93	41.95 ± 2.67	0.693
Platelet (%)	219.50 ± 68.84	226.26 ± 70.73	207.99 ± 64.08	0.007
PDW (fl)	16.15 ± 0.38	16.08 ± 0.37	16.28 ± 0.37	<0.001
Women PDW (fl)	16.08 ± 0.37	16.01 ± 0.35	16.24 ± 0.32	<0.001
Men PDW (fl)	16.28 ± 0.37	16.14 ± 0.37	16.31 ± 0.40	0.003
MPV (fl)	9.94 ± 1.21	9.81 ± 1.17	10.15 ± 1.25	0.005
WBC (10^12^/L)	7.09 ± 2.36	7.14 ± 2.36	7.01 ± 2.37	0.600
Neutrophil (10^12^/L)	4.62 ± 2.00	4.68 ± 2.08	4.52 ± 1.86	0.431
Lymphocyte (10^12^/L)	1.73 (1.35–2.29)	1.73 (1.39–2.29)	1.72 (1.33–2.33)	0.675
**Clotting index**				
D-Dimer (μg/ml)	0.41 (0.23–0.87)	0.40 (0.23–1.06)	0.41 (0.27–0.68)	0.998
Prothrombin time (s)	11.00 ± 0.88	11.02 ± 0.88	10.95 ± 0.89	0.413
**Medication use before admission**				
Antihypertensive drugs (%)	342 (78.08)	209 (75.72)	133 (82.10)	0.120
Lipid lowering drugs (%)	187 (42.69)	80 (28.99)	107 (66.05)	<0.001
Antidiabetic drugs (%)	143 (32.65)	89 (32.25)	54 (33.33)	0.815
Anticoagulant drugs (%)	7 (1.60)	5 (1.81)	2 (1.23)	1.000
Antiplatelet drugs (%)	370 (84.47)	227 (82.25)	143 (88.27)	0.093

*ALT, alanine aminotransferase; CHD, coronary heart disease; ESR, erythrocyte sedimentation rate; HDL-C, high-density lipoprotein cholesterol; LACI, lacunar infarction; LDL-C, low-density lipoprotein; MPV, mean platelet volume; OCSP, oxfordshire community stroke project; PACI, partial anterior circulation infarction; PDW, platelet distribution width; POCI, posterior circulation infarction; RBC, red blood cell; RDW, red blood cell distribution width-standard deviation; TACI, total anterior circulation infarction; WBC, white blood cell*.

### Linear Curve Fitting of the Relationship Between LDL-C and PDW

**Figure 3:**
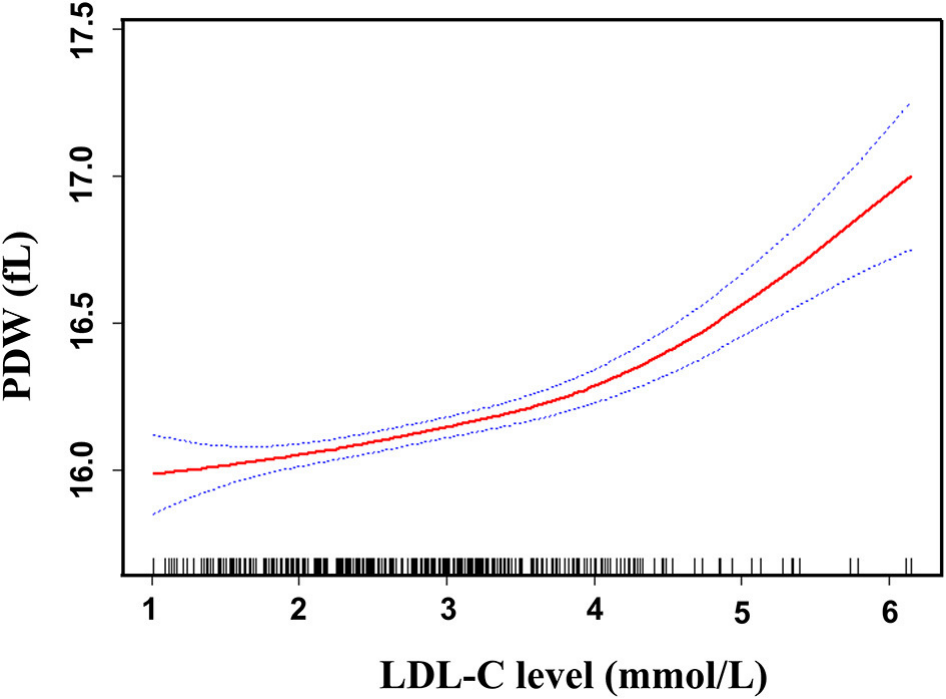
Linear curve fitting of the relationship between LDL-C and PDW. A linear relationship between LDL-C and PDW was detected after adjusting for sex, age, total bilirubin, uric acid, fasting glucose, triglyceride, lipid-lowering drugs, antidiabetic drugs, and antiplatelet drugs. Solid lines represent the fitting curve and dotted lines represent the corresponding 95% CI.

Smoothed plots suggested that there are linear relationships between LDL-C and PDW after adjusting for sex, age, total bilirubin, uric acid, fasting glucose, triglyceride, lipid lowering drugs, antidiabetic drugs, and antiplatelet drugs (Figure 3). Hierarchical analysis by sex also showed there are linear relationships between LDL-C and PDW in women and men (Supplementary Figure 1). The Pearson's correlation coefficients (95%) for the relationship between LDL-C and PDW were 0.438 (0.319–0.543, *p* < 0.001) in women, 0.351 (0.234–0.459, *p* < 0.001) in men, and 0.387 (0.304–0.464, *p* < 0.001) in all patients.

### Multiple Linear Regression Analyses of the Relationship Between LDL-C and PDW

Univariate analysis showed that sex, age, total bilirubin, uric acid, fasting glucose, triglyceride, LDL-C, lipid-lowering drugs, antidiabetic drugs, and antiplatelet drugs were regarded as confounding factors related to PDW (*p* < 0.2, Supplementary Table 2). In multiple linear regression analysis, the beta coefficient (95%) for the relationship between LDL-C and PDW was 0.15 (0.12–0.18, *p* < 0.001) in all patients. When LDL-C levels were divided into normal and high groups, the beta coefficient (95%) for the relationship between LDL-C and PDW was 0.20 (0.13–0.27, *p* < 0.001) reference to normal LDL-C in all patients. When the multivariate analysis was performed after adjusting for sex, age, total bilirubin, uric acid, fasting glucose, triglyceride, lipid-lowering drugs, antidiabetic drugs, and antiplatelet drugs, the beta coefficients (95%) for the relationship between LDL-C and PDW were 0.14 (0.11–0.18, *p* < 0.001) in all patients and 0.18 (0.10–0.25, *p* < 0.001) in the high LDL-C group (Table 2), respectively, which showed that the statistical significance was maintained. Hierarchical analysis, according to sex, age, total bilirubin, uric acid, fasting glucose, triglyceride, lipid-lowering drugs, antidiabetic drugs, and antiplatelet drugs, also showed that the association between LDL-C and PDW was statistically significant (Supplementary Table 3).

**Table 2:** Multivariate linear regression for effects of LDL-C on PDW.

**Variable**	**Model 1 (unadjusted)**		**Model 2 (adjusted)**	
	***N***	**β (95% CI) *p***	***N***	**β (95% CI) *p***
LDL-C (continuous)	438	0.15 (0.12, 0.18) <0.001	438	0.14 (0.11, 0.18) <0.001
LDL-C (categorical)	438		438	
Normal (<3.1 mmol/L)	276	Ref	276	Ref
High (≥3.1 mmol/L)	162	0.20 (0.13, 0.27) <0.001	162	0.18 (0.10, 0.25) <0.001

*Model 1: unadjusted*.

*Model 2: adjusted for sex, age, total bilirubin, uric acid, fasting glucose, triglyceride, lipid-lowering drugs, antidiabetic drugs, and antiplatelet drugs*.

## Discussion

In the current study, we found that elevated LDL-C levels were independently associated with increased PDW in the AIS patients, and this positive effect was evident in all subgroups considered and after careful adjustments. Therefore, these results demonstrated that LDL-C was associated with PDW in AIS patients.

In this study, we observed that higher LDL-C levels were associated with increased levels of PDW, triglyceride, and apolipoprotein B. Dyslipidemia usually includes both elevated LDL-C and triglycerides and reduced HDL-C. Apolipoprotein B is the principal structural apolipoprotein in the LDL-C, and a high LDL-C level is a risk factor for aspirin resistance and cerebrovascular disease (12, 13). Therefore, in our study, we selected LDL-C as the main research object to investigate the interaction between LDL-C and PDW. Oxidized LDL-C and platelet adhesion play an important role in endothelium-mediated vasodilation and hemodynamic changes in the peripheral circulation (14, 15). In 2007, Hsiai and colleagues showed that apolipoprotein B-100 nitration is involved in hemodynamic changes, modulating the focal nature of atherogenesis (16). The treatment of LDL-C apheresis could produce potentially useful hemodynamic effects by significantly reducing the whole blood viscosity at all shear rates (17).

Platelet is the main component of white thrombus, and the elevated PDW increases the risk of platelet adhesion. PDW has been used to distinguish between reactive thrombocytosis and thrombocytosis associated with a myeloproliferative disorder (18). In recent years, PDW is used as a potentially useful marker for the early diagnosis of thromboembolic diseases since it is a specific marker of platelet activation. In 2017, Cetin and colleagues revealed that PDW levels seem to be independent markers of vascular infarction in young patients and may reflect a prothrombotic state (19). Additionally, PDW was applied to evaluate aspirin resistance and coagulation activation efficiently. In 2018, Akturk and colleagues demonstrated that PDW is the determinant of platelet activation and considered it as a marker in inflammatory diseases (20). As mentioned in the above studies, both LDL-C and PDW are involved in platelet adhesion, aspirin resistance, and hemodynamic disorders.

Our study revealed that LDL-C was associated with PDW in AIS patients. While the mechanism of interaction between LDL-C and PDW in AIS is still unclear, it may be related to the oxidative stress response. Increased levels of LDL-C are associated with elevation of oxidized LDL, which promotes the response of oxidative stress and release of inflammatory cytokines (such as functional tissue factor, monocyte chemotactic protein 1, interleukin 10, interleukin 6, and tumor necrosis factor α) in the peripheral circulation (21–24). Oxidized LDL promotes the expression of platelet membrane glycoprotein (CD62P, CD63) and causes the inhibition of the plasma membrane Ca^2+^-ATPase. Its mechanism may be through the pathway of nuclear factor-κB (NF-κB) and signal transducer and activator of transcription 3 (STAT3) (25, 26). In our study, we showed that higher LDL-C levels were associated with increased levels of PDW and uric acid. Previous studies revealed that uric acid is associated with oxidized LDL, and both higher LDL-C and uric acid promote oxidative stress response to produce excessive oxygen free radicals, resulting in the injury of platelet membrane (25, 27, 28). Subsequently, oxidative stress increases the adhesion of platelet, and reduced the deformability of platelet, resulting in elevated PDW (29). In 2012, Badrnya and colleagues showed that platelets accelerate neutrophil transmigration in response to oxidized LDL-C and amplify the inflammatory processes within the vessel wall (30). In 2015, Adam and colleagues demonstrated that LDL-C and PDW were independent predictors of artery stenosis (31). We also observed that patients with anterior circulation cerebral infarction were more than those with posterior circulation in the high-LDL group, which may be related to atherosclerosis and platelet aggregation. Therefore, oxidative stress may be a critical factor for high LDL-C and PDW leading to AIS. In addition, the inflammatory reaction might participate in the reaction between LDL-C and PDW (32). In recent years, biological activity and metabolic pathway of ethanoic acid, prostaenoic acid, phosphocholine, and integrin in the interaction between LDL-C and PDW are being investigated (33–36).

Several limitations should be considered in the interpretation of our results. First, this was a cross-sectional study, and although we did multiple linear regression analysis, establishing a causal link needs further prospective study with a large sample. Second, the study analyzed Chinese AIS patients, the majority of whom were elderly patients with a mean age of about 73 years, and most of the patients were hypertensive. Third, the evidence of interaction between LDL-C and PDW was from relevant literature and needs to be demonstrated by an animal or human experiment. These warrants investigation in other populations to confirm the generalizability of our results. Despite these limitations, in our study, all included patients excluded malignancies and hematological system diseases that may affect the values of PDW, which ensures the reliability of the conclusion.

In conclusion, the present study suggests that LDL-C was associated with PDW in the AIS patients, Therefore, there may be a potential mechanism of LDL-C result in ischemic stroke by changing the homogeneity of platelet volume. Future studies are required to explore the mechanism underlying the association between LDL-C and PDW.

## Data Availability Statement

The raw data supporting the conclusions of this article will be made available by the authors, without undue reservation.

## Ethics Statement

The studies involving human participants were reviewed and approved by Baoshan Branch, Renji Hospital, School of Medicine, Shanghai Jiaotong University, Shanghai. The patients/participants provided their written informed consent to participate in this study. Written informed consent was obtained from the individual(s) for the publication of any potentially identifiable images or data included in this article.

## Author Contributions

D-SZ, JYu, and Z-ZL analyzed data and wrote the manuscript. PZ, R-HH, JD, JYa, NZ, and X-JZ performed the data curation. JC and Q-LF supervised the data curation. JZ conducted formal analysis. Y-TG and D-SZ supervised this research. All authors read and approved the final manuscript.

## Conflict of Interest

The authors declare that the research was conducted in the absence of any commercial or financial relationships that could be construed as a potential conflict of interest.

## Supplementary Material

Supplementary Figure 1 Linear curve fitting of the relationship between LDL-C and PDW by sex. Hierarchical analysis by sex also showed there are linear relationships between LDL-C and PDW in women and men after adjusting for confounder factors.
