# Supplementary figures and images for: Association Between Low-Density Lipoprotein Cholesterol and Platelet Distribution Width in Acute Ischemic Stroke
Source: Front Neurol. 2021 Mar 5;12:631227. doi: 10.3389/fneur.2021.631227 (PMC7973264; doi:10.3389/fneur.2021.631227)

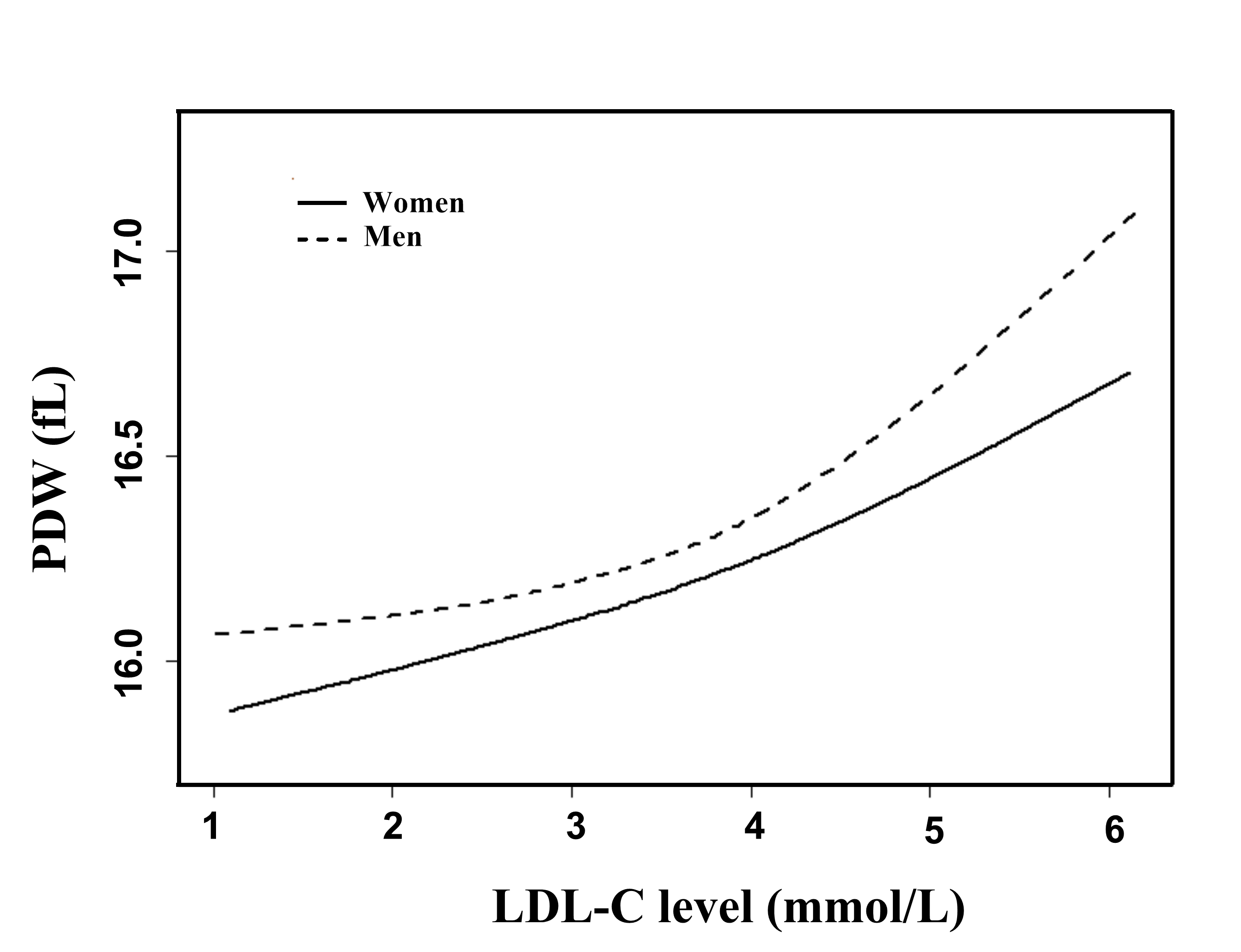

Supplement: Supplementary Figure 1 — Linear curve fitting of the relationship between LDL-C and PDW by sex. Hierarchical analysis by sex also showed there are linear relationships between LDL-C and PDW in women and men after adjusting for confounder factors. [file Image_1.tif]
