# Supplementary material for: Association Between Low-Density Lipoprotein Cholesterol and Platelet Distribution Width in Acute Ischemic Stroke
Source: Front Neurol. 2021 Mar 5;12:631227. doi: 10.3389/fneur.2021.631227 (PMC7973264; doi:10.3389/fneur.2021.631227)
Supplement: Supplementary file 2 [file Table_1.docx]

**Supplement Table1.** Comparison of PDW by the tertiles of LDL-C levels

| **Variables** | **LDL-C levels** | | | ***p*-value** |
| --- | --- | --- | --- | --- |
|  | **Tertile 1 (Low)** | **Tertile 2 (Middle)** | **Tertile 3 (High)** |  |
| **N** | 143 | 149 | 146 |  |
| LDL-C (mmol/L) (min-max) | 1.01-2.27 | 2.28-3.19 | 3.20-6.15 | <0.001 |
| All patients PDW (fL) | 16.03±0.40 | 16.14±0.32 | 16.29±0.37 | <0.001 |
| Women PDW (fL) | 15.95 ± 0.38 | 16.09 ± 0.29 | 16.25 ± 0.32 | <0.001 |
| Men PDW (fL) | 16.11 ± 0.41 | 16.18 ± 0.33 | 16.32 ± 0.41 | 0.003 |
