# Supplementary material for: Association Between Low-Density Lipoprotein Cholesterol and Platelet Distribution Width in Acute Ischemic Stroke
Source: Front Neurol. 2021 Mar 5;12:631227. doi: 10.3389/fneur.2021.631227 (PMC7973264; doi:10.3389/fneur.2021.631227)
Supplement: Supplementary file 3 [file Table_2.docx]

**Supplement Table 2.** **Univariate analysis of PDW**

| **Variable** | **Statistics** | **β (95% CI)** | ***p* value** |
| --- | --- | --- | --- |
| Sex (%) |  |  |  |
| Women | 202 (46.12) | ref |  |
| Men | 236 (53.88) | 0.11 (0.04, 0.19) | 0.001 |
| Age (years) | 73.07±10.83 | -0.01 (-0.02, 0.00) | 0.192 |
| Total bilirubin (μmol/L) | 13.17±7.58 | 0.01 (0.00, 0.01) | 0.004 |
| Uric acid (μmol/L) | 291.31±109.11 | 0.01 (0.00, 0.01) | 0.019 |
| Fasting glucose (mmol/L) | 6.16±2.20 | 0.03 (0.01, 0.04) | 0.001 |
| Triglyceride (mmol/L) | 1.35±0.89 | 0.08 (0.04, 0.12) | <0.001 |
| LDL-C (mmol/L) | 2.84±0.98 | 0.15 (0.12, 0.18) | <0.001 |
| Lipid lowering drugs (%) |  |  |  |
| No | 251 (57.31) | ref |  |
| Yes | 187 (42.69) | 0.08 (0.01, 0.15) | 0.029 |
| Antidiabetic drugs (%) |  |  |  |
| No | 295 (67.35) | ref |  |
| Yes | 143 (32.65) | 0.08 (0.00, 0.15) | 0.042 |
| Antiplatelet drugs (%) |  |  |  |
| No | 68 (15.53) | ref |  |
| Yes | 370 (84.47) | -0.02 (-0.04, 0.00) | 0.137 |
