# Supplementary material for: Association Between Low-Density Lipoprotein Cholesterol and Platelet Distribution Width in Acute Ischemic Stroke
Source: Front Neurol. 2021 Mar 5;12:631227. doi: 10.3389/fneur.2021.631227 (PMC7973264; doi:10.3389/fneur.2021.631227)
Supplement: Supplementary file 4 [file Table_3.docx]

**Supplement Table 3.** Hierarchical analysis on relationship of LDL-C and PDW

| **Hierarchical factor** | | N | **β (95% CI) *p*** | ***p* for interaction** |
| --- | --- | --- | --- | --- |
| Sex | Women | 202 | 0.15 (0.10, 0.19) <0.001 | 0.835 |
|  | Men | 236 | 0.14 (0.09, 0.19) <0.001 |  |
| Age (years) | <75 | 244 | 0.14 (0.09, 0.19) <0.001 | 0.794 |
|  | ≥75 | 194 | 0.15 (0.10, 0.20) <0.001 |  |
| Total bilirubin (μmol/L) | <22.25 | 405 | 0.14 (0.10, 0.17) <0.001 | 0.131 |
|  | ≥22.25 | 33 | 0.23 (0.11, 0.35) <0.001 |  |
| Uric acid (μmol/L) | <426 | 386 | 0.15 (0.12, 0.19) <0.001 | 0.183 |
|  | ≥426 | 52 | 0.09 (-0.01, 0.18) 0.081 |  |
| Fasting glucose (mmol/L) | <6.1 | 281 | 0.16 (0.12, 0.21) <0.001 | 0.185 |
|  | ≥6.1 | 157 | 0.12 (0.06, 0.17) <0.001 |  |
| Triglyceride (mmol/L) | <1.7 | 345 | 0.15 (0.11, 0.19) <0.001 | 0.321 |
|  | ≥1.7 | 93 | 0.11 (0.03, 0.18) 0.007 |  |
| Lipid lowering drugs | No | 251 | 0.12 (0.06, 0.17) <0.001 | 0.126 |
|  | Yes | 187 | 0.17 (0.12, 0.22) <0.001 |  |
| Antidiabetic drugs | No | 295 | 0.14 (0.10, 0.18) <0.001 | 0.790 |
|  | Yes | 143 | 0.15 (0.09, 0.21) <0.001 |  |
| Antiplatelet drugs | No | 68 | 0.10 (0.02, 0.18) 0.010 | 0.239 |
|  | Yes | 370 | 0.15 (0.11, 0.19) <0.001 |  |

Each stratification adjusted for all the factors (sex, age, total bilirubin, uric acid, fasting glucose, triglyceride, lipid lowering drugs, antidiabetic drugs and antiplatelet drugs) except the stratification factor itself.
